# Prognostic factors of a lower CD4/CD8 ratio in long term viral suppression HIV infected children

**DOI:** 10.1371/journal.pone.0220552

**Published:** 2019-08-05

**Authors:** Sara Guillén, Luis Prieto, Santiago Jiménez de Ory, María Isabel González-Tomé, Pablo Rojo, María Luisa Navarro, María José Mellado, Luis Escosa, Talía Sainz, Laura Francisco, María Ángeles Muñoz-Fernández, José Tomás Ramos, On behalf of CoRISpe (Cohorte Nacional de VIH pediátrica de la RED RIS)

**Affiliations:** 1 Department Pediatrics, Hospital Universitario de Getafe, Madrid, Spain; 2 Department of Pediatrics, Hospital Universitario 12 de Octubre, Madrid, Spain; 3 Department of Immunology, Hospital Universitario Gregorio Marañón, Madrid, Spain; 4 Department of Pediatrics, Hospital Universitario Gregorio Marañón, Madrid, Spain; 5 Department of Pediatrics, Hospital Universitario La Paz, Madrid, Spain; 6 Department of Pediatrics, Hospital Clínico Universitario San Carlos, Madrid, Spain; Azienda Ospedaliera Universitaria di Perugia, ITALY

## Abstract

**Background:**

Combination antiretroviral therapy (cART) is associated with marked immune reconstitution. Although a long term viral suppression is achievable, not all children however, attain complete immunological recovery due to persistent immune activation. We use CD4/CD8 ratio like a marker of immune reconstitution.

**Methods:**

Perinatal HIV-infected children who underwent a first-line cART, achieved viral suppression in the first year and maintained it for more than 5 years, with no viral rebound were included. Logistic models were applied to estimate the prognostic factors, clinical characteristics at cART start, of a lower CD4/CD8 ratio at the last visit.

**Results:**

146 HIV-infected children were included: 77% Caucasian, 45% male and 28% CDC C. Median age at cART initiation was 2.3 years (IQR: 0.5–6.2). 42 (30%) children received mono-dual therapy previously to cART. Time of undetectable viral load was 9.5 years (IQR: 7.8, 12.5). 33% of the children not achieved CD4/CD8 ratio >1. Univariate analysis showed an association between CD4/CD8 <1 with lower CD4 nadir and baseline CD4; older age at diagnosis and at cART initiation; and a previous exposure to mono-dual therapy. Multivariate analysis also revealed relationship between CD4/CD8 <1 and lower CD4 nadir (OR: 1.002, CI 95% 1.000–1.004) as well as previous exposure to mono-dual therapy (OR: 0.16, CI 95% 0.003–0.720).

**Conclusions:**

CD4/CD8 >1 was not achieved in 33% of the children. Lower CD4 nadir and previous exposure to suboptimal therapy, before initiating cART, are factors showing independently association with a worse immune recovery (CD4/CD8 < 1).

## Introduction

The combination antiretroviral therapy (cART) has led not only to a decrease in HIV viral load but also to the recovery of CD4 T cell count and ultimately to the clinical improvement of HIV-infected children [1, 2]. Plasma viremia suppression by cART is generally associated with improvements in the immunological condition of patients, but some children fail to achieve normalization of CD4 T cell count. [3]

Various mechanisms such as immune activation, on-going HIV replication and deficient thymus output are associated with impaired immunological response to treatment in adults. [4] In vertically HIV-1 infected children, the infection is acquired when the immune system is still immature and the thymus is activated to produce new naïve T cells. [2] Highly active thymus in early childhood may contribute to a better immune reconstitution if ART is initiated early in life. [5] With the increase in age, the recovery through the naïve pool is progressively damaging, hence early treatment is the key for immune reconstitution. [6] However, other studies suggest that the recovery of the thymic function is age-independent and it is related to peripheral CD4 cell depletion as well as HIV-1 suppression. [7]

Little is known about long term immune recovery in HIV-infected children on cART. Immune recovery has been associated with earlier age, higher CD4 cell count and CD4 nadir at cART initiation, whereas, severe immunosuppression is associated with an impaired CD4 recovery.[8, 9, 10, 11] cART significantly reduces the degree of immune activation and detectable viral load aggravates immune senescence in children. [12, 13]

Restoration of CD4/CD8 ratio requires both CD4 cell recovery and normalization of the CD8 count. CD4/CD8 ratio has been associated with T cell activation, despite long term viral suppression in both, children and adults, as well as evidence to support that this imbalance has clinical importance in children. [14, 15, 16] A persistently low CD4/CD8 ratio despite viral control reflects a higher risk of morbidity in HIV-infected adults. [17] In addition, CD4/CD8 ratio has been described as a predictor of CD4 response in HIV-1-infected adults. [18] So we used CD4/CD8 ratio as a better predictor of long term immune recovery, since CD4 does not predict immune activation.

The aim of this study was to study possible risk factors of not achieving a CD4/CD8 >1 in children with long term immune reconstitution and maintained undetectable viral load, in order to determine possible risk factors that could affect the immunological response to cART.

## Materials and methods

### Study design

IRB (Hospital de Getafe) approved the study. This is a multicenter observational study, within the Pediatric branch of the National AIDS Research Network of Spain (CoRISpe). 383 perinatal HIV-infected children were in follow up in this cohort when the study was conducted. We included children who met the following inclusion criteria: 1) were receiving cART when the study was conducted; 2) who underwent first-line cART, had evidence of virological suppression within the first year after cART initiation; 3) had subsequent maintenance for more than 5 years with no viral rebound longer than 3 months at each time or at least until last observation before transition to adult units. 146 children were analyzed. The study was conducted from 1997 to December 2015.

Data were retrospectively collected from clinical visits performed every 3 months since cART initiation. A duly signed informed consent was obtained from the parents or legal guardians of patients <12 years and direct informed assent from patients aged ≥12 years in order to participate in CoRISpe.

### Definitions

First-line cART refers to those patients that had not taken any cART regimen previously, but could have taken mono or dual antiretroviral therapy. Viral suppression was defined as <400 HIV-RNA copies/ml.

Patients with a CD4/CD8 ratio >1 at last clinical visit, were considered to have achieved immune recovery and patients with a CD4/CD8 < 1 were considered not to reach immune recovery. We studied possible risk factors at baseline of not achieving a CD4/CD8 >1 at last visit, comparing both groups.

We considered baseline time when children started first line of cART.

### Statistics

All continuous variables were expressed as median (IQR), and categorical variables as frequency of distribution and rates. Differences between categorical variables were analysed using the Chi square test and Fisher exact test. T student test was used to compare continuous variables. A multivariable logistic regression model was used to analyse the relationship between immune recovery and variables showing statistical significance in univariate analysis. Data were analyzed using IBM SPSS Statistics for Windows, Version 21.0 (Armonk, NY: IBM Corp). A P value of 0.05 was considered statistically significant.

## Results

### Clinical characteristics at baseline

A total of 146 HIV-infected children were enrolled in this study. Results are shown in Table 1. In general, 81 (55%) were female and 47 (33%) were non Caucasian. Their median age at diagnosis was 0.6 years (IQR: 0.2, 2.1), the median age at cART initiation was 2.3 years (IQR: 0.5, 6.2) and 19 (28%) were on CDC stage C. Mono-dual therapy was taken by 42 (30%) patients previous to cART, the rest of patients were naïve. The median of CD4 nadir was 457 cells/mm^3^ (IQR: 269, 678) and the median of CD4 cells and CD4% at baseline were 901 (IQR: 470, 1800) cells/mm^3^ and 26% (IQR: 16, 35), respectively. The median CD4/CD8 ratio at baseline was 0.6 (IQR: 0.3, 1.2).

**Table 1:** Baseline clinical characteristics of subjects.

Characteristics	Total
**N**	146
**Female gender, n (%)**	81 (55%)
**Age at diagnosis (years), median (IQR)**	0.6 (0.2, 2.1)
**Age at HAART initiation, median (IQR)**	2.3 (0.5, 6.2)
**Age at the last visit, median (IQR)**	14.4 (10.6, 18.1)
**Ethnicity, n (%)**	
Caucasian	97 (67%)
Non Caucasian	47 (33%)
**Clinical stage at baseline, n (%)**	
N/A	78 (53.5%)
B	40 (27.5%)
C	19 (28%)
**Nadir CD4 (cells/mm^3^), median (IQR)**	457 (269, 678)
**Nadir CD4%, median (IQR)**	19 (11, 25)
**Prior history of ART, n (%)**	
Mono or dual ART	42 (30%)
Naïve	104 (70%)
**cART**	
Total n° of ART regimens, median (IQR)	3 (2, 5)
Total n° of cART regimens, median (IQR)	2 (2, 4)
Duration of cART (years), median (IQR)	9.8 (8.1, 13.2)
**Baseline CD4 and CD8**	
CD4 (cells/mm^3^) at baseline, median (IQR)	901 (470, 1800)
CD4% at baseline, median (IQR)	26 (16, 35)
CD8 (cells/mm^3^) at baseline, median (IQR)	1398 (861, 1964)
CD8% at baseline, median (IQR)	37 (28, 40.5)
CD4/CD8 ratio at baseline, median (IQR)	0.6 (0.3, 1.2)
**Viral load**	
Viral load at baseline (log_10_), median (IQR)	4.88 (4.12, 5.67)
Time of undetectable viral load, median (IQR)	9.5 (7.8, 12.5)

### Follow up and immune recovery

As shown in Table 1, the median follow up period of patients in this study was more than 10 years, with a median duration of cART of 9.8 years (IQR: 8.1, 13.2) and a median of 2 different cART regimens taken per patient (IQR: 2, 4). The patients remained with undetectable viral load for 9.5 years (IQR: 7.8, 12.5). 63 of 146 children (43%) had a viral blip in the follow up. Median age at last observation was 14.4 years (IQR: 10.6, 18.1).

In general, the median CD4 count and percentage at last visit were 878 cells/mm^3^ (IQR: 672, 1085) and 38% (IQR: 33, 43), respectively, and the median CD4 /CD8 ratio was 1.2 (IQR: 0.9, 1.6).

### Factors associated with not immune recovery

At last clinical visit, immune recovery (CD4/CD8 ratio >1) was not achieved in 48/146 (33%) children. Results are shown in Table 2.

**Table 2:** Prognostic factors of subjects by immune recovery.

	Univariate analysis			Multivariate analysis	
Characteristics	Non Immune recovery CD4 /CD8 < 1	Immune recovery CD4/CD8 > 1	p	Adjusted difference Odds ratio (95% CI)	p
**N**	48	98	-		
**Female gender, n (%)**	26 (32)	55 (68)	0,8		
**Age at diagnosis (years), median (IQR)**	1.4 (0.2, 3.6)	0.5 (0.1–1.8)	0.03	0.957 (0.745–1.231)	0.7
**Age at HAART initiation, median (IQR)**	5.6 (2.1, 8.06)	1.3 (0.3, 4.4)	< 0.001	0.951 (0.789–1.147)	0.6
**Age at the last visit, median (IQR)**	16.8 (13.4, 18.7)	13.5 (10.1. 17.8)	0.01		
**Ethnicity, n (%)**					
Caucasian	33	64	0.7		
Non Caucasian	14	33			
**Clinical stage at baseline, n (%)**					
N/A	25	53	0.6		
B	12	26			
C	10	18			
**Nadir CD4 (cells/mm^3^), median (IQR)**	323 (153, 503)	543 (350, 764)	< 0.001	1.002 (1.000–1.004)	0.01
**Nadir CD4%, median (IQR)**	14.5 (8–21)	21.5 (14–28)	< 0.001		
**Prior history of ART, n (%)**					
Mono or dual ART	23	19	0.001	0.164 (0.037–0.725)	0.01
Naïve	25	79			
**cART median (IQR)**					
Total n° of ART regimens	4 (3, 5)	3 (2, 4)	0.2		
Total n° of cART regimens	2 (2, 3)	3 (2, 4)	0.5		
Duration of cART (years)	9.5 (7.4–12)	10.6 (8.2–13.8)	0.1		
**Baseline CD4 and CD8 median (IQR)**					
CD4 (cells/mm^3^) at baseline	563 (201, 1039)	1264 (735, 2299)	<0.001	1.000(0.999–1.001)	0.5
CD4% at baseline	20 (8, 28)	32 (20, 38)	<0.001		
CD8 (cells/mm^3^) at baseline	1411 (866, 1933)	1375 (861–1964)	0.61		
CD8% at baseline	48 (38–60)	35 (22–44)	<0.001		
CD4/CD8 ratio at baseline	0.4 (0.1, 0.6)	0.9 (0.5, 1.6)	<0.001	1.777 (0.782–4.035)	0.1
**Viral load median (IQR)**					
Viral load at baseline (log_10_)	4.8 (4, 5.4)	4.9 (4.2, 5.7)	0.4		
Time of undetectable viral load	9.1 (7.7, 11.4)	9.8 (7.9–12.6)	0.2		

When comparing both groups of patients, the significant predictors of not immune recovery by univariate analyses were lower CD4 nadir, lower CD4 count and percentage, lower CD4/CD8 ratio and higher CD8 percentage at baseline.

An older age at HIV diagnosis and cART initiation was also associated to a unsuccessful immune response. Being exposed to mono-dual therapy was also a predictor for worse immune recovery.

In multivariate analysis, a CD4/CD8 ratio < 1 was associated with lower CD4 nadir, (OR: 1.002, CI 95% 1.000–1.004) as well as previous exposure to mono-dual therapy (OR: 0.16, CI 95% 0.003–0.720).

## Discussion

This study described the immune response in HIV-infected children treated with long term cART, and reported an independent association between the lower CD4 nadir of patients and the exposure to suboptimal ART regimens with a CD4/CD8 ratio <1, which represents a unsuccessful immune recovery.

CD4/CD8 > 1 was not achieved in our study in 33% of the patients. Although the majority of the studies in children define immune recovery as a CD4 percentage greater than 25% [19, 20], we used CD4/CD8 ratio as a better predictor of long term immune recovery, since CD4 does not predict immune activation. Recently, the CD4/CD8 ratio has been described as a marker of immune activation in virologically suppressed HIV adults and children patients. [14, 15, 16, 21] Moreover, the CD4/CD8 ratio can contribute to the immunological evaluation of treated patients in a long-term follow-up and may be applied for monitoring both immune dysfunction and viral reservoir size in virologically suppressed HIV-positive adults. [22]

The median follow-up period of patients on cART in this study was nearly 10 years. To our knowledge, this study is the largest to date in terms of long term follow-up period on immune recovery in children. Other studies, however, showed a follow-up period of 5 to 7 years in naïve children and a follow-up period of 6 years in pre-treated children. [1, 19, 20]

We did not find any association between cART duration or number of regimens and immune recovery, although was nearly significant (p value was 0.1 and p 0.2 respectively), probably due to our small sample size. Both groups received a long term therapy with a median duration of nearly 10 years with various regimens. A longer duration of cART was an independent predictor of immune recovery in a study from Thailand. [19] We did not compare the immune recovery between the different antiretroviral drugs used, but it was described that in adults, normalization of the CD4/CD8 ratio above a clinically meaningful threshold may be dependent on the drug class used. [23]

Receiving suboptimal treatment such as mono-dual therapy could lead to an impaired immune recovery, because these therapies were not efficacy to achieve persistent undetectable viral load and immune recovery. So initiating cART, after severe immunosuppression has occurred, is detrimental for the restoration of the CD4 cell count. A third of the patients in our study had previously received mono or dual therapy and, therefore, achieved a lower CD4/CD8 ratio at the end of the study compared to naïve patients. Our results are consistent with those in a study covering a similar population, except that all subjects had previously received mono or dual therapy where they found a significant association between not reaching CD4% > 25% during follow-up and the duration of antiretroviral therapy before cART. [1]

A worse immune recovery in our study, while comparing the two groups, was associated with older age at diagnosis and at cART initiation in univariate analysis. Our interpretation to this, as reported in previous studies [8, 19, 24, 25, 26], is that early initiation of cART may improve the immune recovery. However, these associations in our study were not maintained in multivariate analysis. Data from few studies suggested that the immune recovery is independent of the age at cART initiation. [9, 27] Better viral suppression but poorer immunological responses have been described in older individuals compared to children. [10] The median time to achieve immunological response (CD4> 30%) has been described to be shorter in children younger than two years, irrespective of pre-cART CD4 counts and the time to immune recovery, and it increased progressively in older children. [25]

Initiation of cART after severe immunosuppression was described as ineffective in several studies for a normal CD4 count recovery. [1, 11] A study conducted in the United States showed that children who started ART and who had a higher nadir CD4% were more likely to achieve a higher CD4%. [8] Two studies conducted in Thailand showed that higher baseline CD4% was associated with better achievement of immune recovery. [28, 29] These results show consistency with the ones observed in our cohort, as univariate analysis showed that patients who had worse immune recovery had a lower CD4 nadir and a lower CD4 count at baseline, although, only the CD4 nadir remained independently associated to immune reconstitution in multivariate analysis. Other studies, have described that patients under greater immunosuppression had the largest CD4 increase, but only a minority of these patients achieved CD4 > 25%. [20, 24]

So we can say, like in other studies in adults that early initiation, before CD4 decrease, and effective ART appear to improve CD4/CD8 ratio in long term viral suppression patients. [14]

CDC clinical stage was not associated with immune recovery in our study. In the European Collaborative Cohort study, children on clinical stage C were less likely to achieve a 20% increase in CD4 z-score, compared to those who had cART initiated at clinical stage B or A. [26] In two studies conducted in Thailand, clinical CDC stage was not an independent predictor for reaching CD4 percentage > 25% after cART initiation. [19, 28]

In our study, higher baseline CD8 counts and lower baseline CD4/CD8 ratio were associated with worse immune recovery in univariate analysis. Baseline absolute CD8 cell counts and CD4/CD8 ratio has been described influencing the recovery of CD4/CD8 ratio and CD4 response in HIV-infected adults. [18, 30, 31]

No associations were found between baseline viral load and immune recovery in our population, which is consistent with other published data. [19] Longer duration of undetectable viral load was nearly significant to achieve a CD4/CD8 >1, with p value 0.2, probably due to the small size of the sample.

Moreover, in our study, ethnicity could not be considered as a predictor for immune response, as also reported in a study conducted in children in The Netherlands, despite initial differences in CD4 counts. [32] Similar results were found in adult population, observing a significantly lower CD4/CD8 ratio prior to commencing ART in Asian patients compared to Caucasian, but after adjustment, there was no significant difference between the cohorts in odds of achieving normal ratio. [33]

A limitation of this study is that, despite being the largest study to date in terms of the length of follow-up period of patients; in describing the immune reconstitution, the sample size is not representative of the entire Spanish cohort due to the fact that there were missing data for most patients in such a long follow-up period and, also due to the observational nature of the study. The second limitation is that CD4/CD8 ratio is an imperfect measure of immune reconstitution and immune activation and we did not consider other laboratory parameters that are now commonly used to describe immune reconstitution (quantification of CD31+CD45RA+CD4+ T cells) or immune activation (HLADR CD38 expression). The third limitation is that 30% of children received mono or dual therapy, which implies that these children started therapy in an earlier era of treatment when less potent antiretroviral therapy were available and this potential bias was not addressed.

## Conclusion

This study showed the long-term immune recovery after 10 years of initiating and maintaining suppressive cART, nonetheless one third of patients did not achieve a CD4/CD8 ratio > 1, being associated with a lower CD4 nadir and receiving previously mono or dual therapy. All these data suggest that there is a difficulty in achieving immune recovery after severe immune deterioration, which highlights the importance of an early and effective treatment initiation. In addition, our observations showed a relationship between incomplete immune recovery and immune activation, despite maintained undetectable viral load, which requires longer follow up to elucidate its potential long term consequences.
